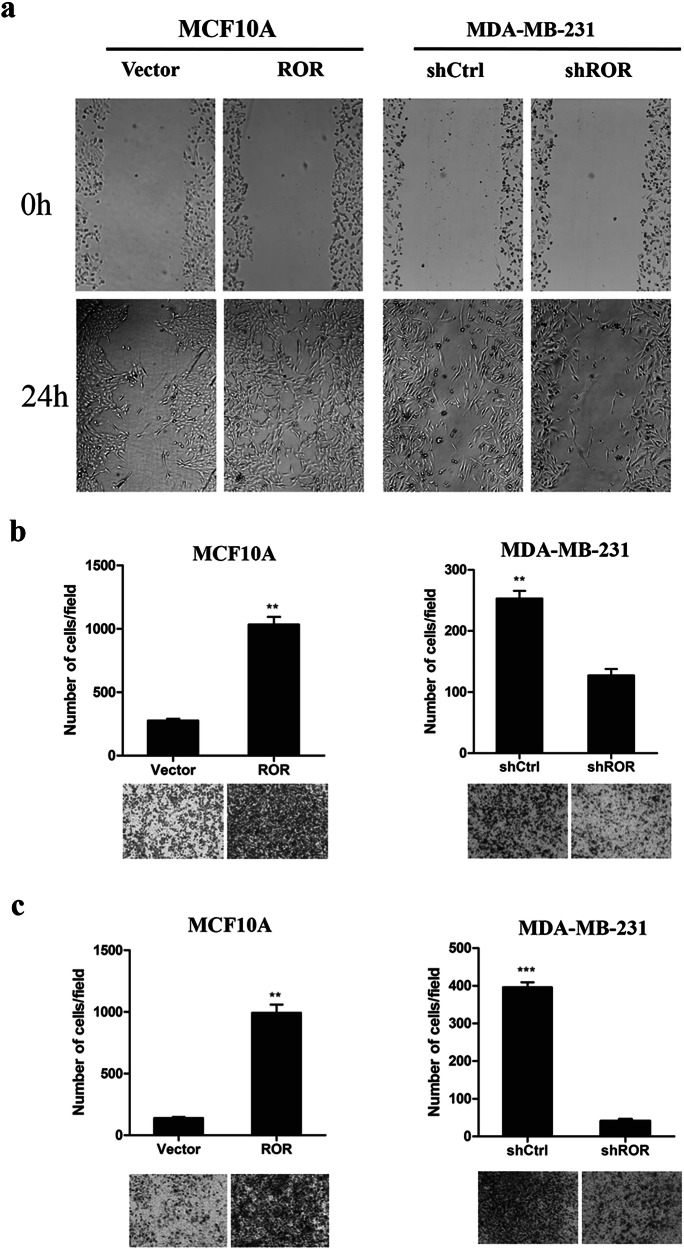# Correction: LincRNA-ROR induces epithelial-to-mesenchymal transition and contributes to breast cancer tumorigenesis and metastasis

**DOI:** 10.1038/s41419-025-07683-0

**Published:** 2025-04-25

**Authors:** P. Hou, Y. Zhao, Z. Li, R. Yao, M. Ma, Y. Gao, L. Zhao, Y. Zhang, B. Huang, J. Lu

**Affiliations:** 1 The Institute of Genetics and Cytology, Northeast Normal University, Changchun, China; 2 The Key Laboratory of Molecular Epigenetics of Ministry of Education (MOE), Changchun, China; 3 The Breast Surgery, The Tumor Hospital of Jilin Province, Changchun, China

Correction to: *Cell Death and Disease* 10.1038/cddis.2014.249, published online 12 June 2014

Dear editor:

We noticed that there is an error in our manuscript. “LincRNA-ROR induces epithelial-to-mesenchymal transition and contributes to breast cancer tumorigenesis and metastasis.” Cell Death Dis 5, e1287 (2014) 10.1038/cddis.2014.249. After checking the raw data, we want to provide the correct version.

In preparing the figures of our paper, we did insert the wrong image of the representative migration cells image of the vector group unintentionally in figure 3b. We apologize for the error and for any inconvenience that it may have caused. The corrected version of Figure 3b now includes the correct image, the replaced image was marked using red frame. Also, the raw data of the figure will be provided if required.

We apologize for the error and for any inconvenience that may have caused, and confirm that these errors did not affect the conclusions reported in the paper.

**Published Figure 3B** 
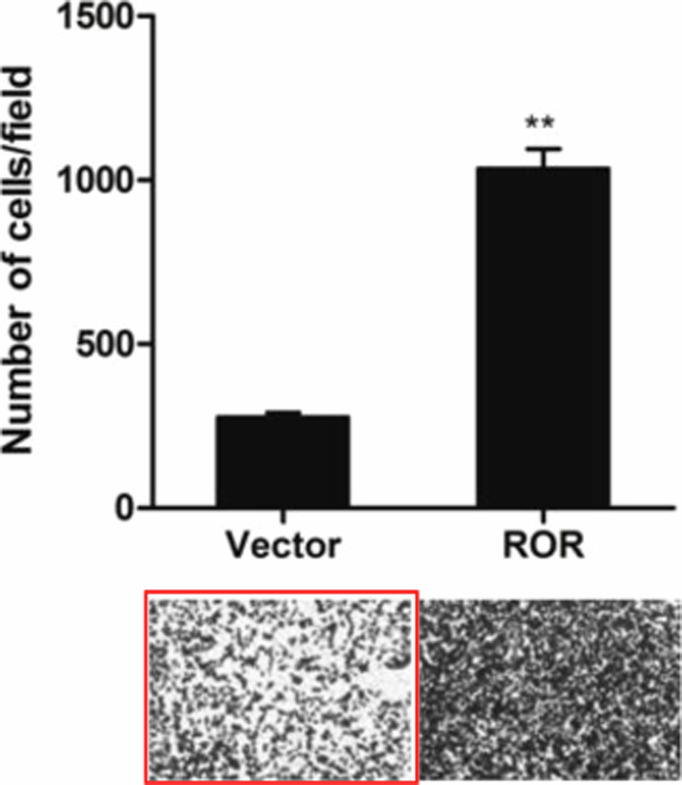


**Corrected Figure 3B** 
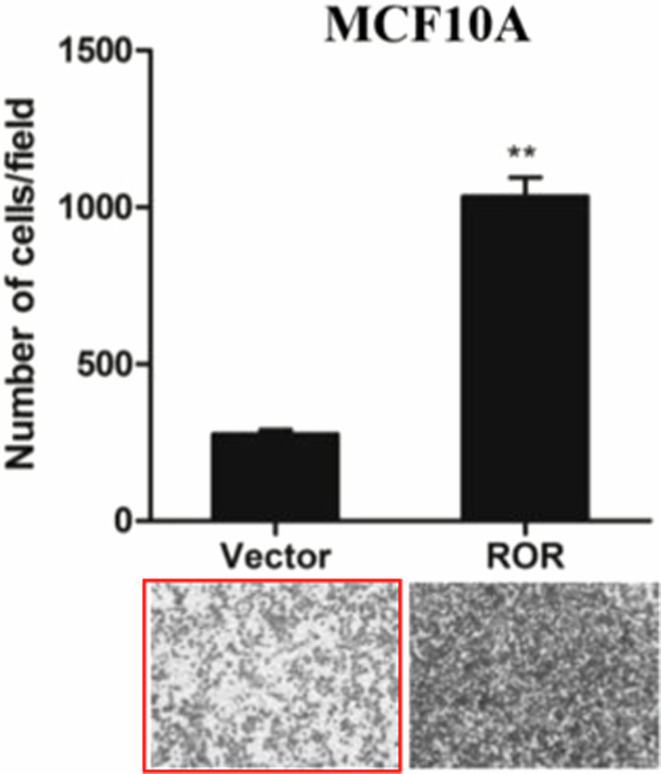


**Corrected Fig 3**